# Analysis of mRNA and Protein Levels of *CAP2*, *DLG1* and *ADAM10* Genes in Post-Mortem Brain of Schizophrenia, Parkinson’s and Alzheimer’s Disease Patients

**DOI:** 10.3390/ijms23031539

**Published:** 2022-01-28

**Authors:** Anna Di Maio, Arianna De Rosa, Silvia Pelucchi, Martina Garofalo, Benedetta Marciano, Tommaso Nuzzo, Fabrizio Gardoni, Andrea M. Isidori, Monica Di Luca, Francesco Errico, Andrea De Bartolomeis, Elena Marcello, Alessandro Usiello

**Affiliations:** 1 Laboratory of Translational Neuroscience, CEINGE Biotecnologie Avanzate, 80145 Naples, Italy; dimaio@ceinge.unina.it (A.D.M.); derosaar@ceinge.unina.it (A.D.R.); garofalom@ceinge.unina.it (M.G.); marcianob@ceinge.unina.it (B.M.); nuzzo@ceinge.unina.it (T.N.); francesco.errico@ceinge.unina.it (F.E.); 2 Department of Experimental Medicine, Sapienza University of Rome, 00185 Rome, Italy; andrea.isidori@uniroma1.it; 3 Department of Pharmacological and Biomolecular Sciences (DiSFeB), University of Milan, 20133 Milan, Italy; silviapelucchi87@gmail.com (S.P.); fabrizio.gardoni@unimi.it (F.G.); monica.diluca@unimi.it (M.D.L.); 4 Department of Environmental, Biological and Pharmaceutical Science and Technologies, Università degli Studi della Campania “Luigi Vanvitelli”, 81100 Caserta, Italy; 5 Department of Agricultural Sciences, University of Naples “Federico II”, 80055 Naples, Italy; 6 Section of Psychiatry Laboratory of Molecular and Translational Psychiatry, Department of Neuroscience, Reproductive Science and Odontostomatology, School of Medicine, University “Federico II”, 80131 Naples, Italy; adebarto@unina.it

**Keywords:** dendritic spine, postsynaptic density, schizophrenia, Alzheimer’s disease, Parkinson’s disease

## Abstract

Schizophrenia (SCZ) is a mental illness characterized by aberrant synaptic plasticity and connectivity. A large bulk of evidence suggests genetic and functional links between postsynaptic abnormalities and SCZ. Here, we performed quantitative PCR and Western blotting analysis in the dorsolateral prefrontal cortex (DLPFC) and hippocampus of SCZ patients to investigate the mRNA and protein expression of three key spine shapers: the actin-binding protein cyclase-associated protein 2 (*CAP2*), the sheddase a disintegrin and metalloproteinase 10 (*ADAM10*), and the synapse-associated protein 97 (*SAP97*). Our analysis of the SCZ post-mortem brain indicated increased *DLG1* mRNA in DLPFC and decreased *CAP2* mRNA in the hippocampus of SCZ patients, compared to non-psychiatric control subjects, while the *ADAM10* transcript was unaffected. Conversely, no differences in *CAP2*, *SAP97*, and *ADAM10* protein levels were detected between SCZ and control individuals in both brain regions. To assess whether *DLG1* and *CAP2* transcript alterations were selective for SCZ, we also measured their expression in the superior frontal gyrus of patients affected by neurodegenerative disorders, like Parkinson’s and Alzheimer’s disease. Interestingly, also in Parkinson’s disease patients, we found a selective reduction of *CAP2* mRNA levels relative to controls but unaltered protein levels. Taken together, we reported for the first time altered *CAP2* expression in the brain of patients with psychiatric and neurological disorders, thus suggesting that aberrant expression of this gene may contribute to synaptic dysfunction in these neuropathologies.

## 1. Introduction

Schizophrenia (SCZ) is a polygenic and multifactorial disorder with complex phenotypes encompassing multiple domains, such as delusions and hallucinations (positive symptoms), avolition, anhedonia, lack of social interaction (negative symptoms), and deficit of executive functions (cognitive symptoms) [1,2]. SCZ has been conceptualized as a neurodevelopmental disorder [3,4] affecting synaptic plasticity [4] and cortical–subcortical connectome. Dendritic spines are critical elements of brain circuits since they establish most excitatory synapses. The tip of dendritic spines contains a disk-shaped structure, named the postsynaptic density (PSD), that is believed to be deranged in SCZ [5,6]. The PSD is constituted of approximately 1500 molecules including glutamate receptors, scaffolding proteins, adhesion molecules, and enzymes. Therefore, the PSD has also been recognized as a structural and functional hub [7], responsible for postsynaptic signaling and synaptic plasticity. The molecules of the PSD are believed to be grouped in nanoclusters around a few proteins and organized dynamically by phase separation [8]. Dendritic spines are highly dynamic elements and have the capacity to undergo structural changes that are tightly coordinated with synaptic function and modifications in glutamate receptors [9]. Remarkably, the morphological alterations of spines are considered as the structural basis for learning and encoding memories [10,11]. In line with the glutamatergic hypothesis of SCZ, a significant reduction in spine density has been identified within the dorsolateral prefrontal cortex (DLPFC) and hippocampus of patients affected by this mental illness [12]. This view is reinforced by structural imaging studies of SCZ patients showing smaller whole brain volumes, such as a reduction of the prefrontal cortex (PFC) [13,14] and the hippocampus [15,16]. The essential elements that control dendritic spine shape and its remodeling in response to plasticity are the actin cytoskeleton [17] and the cell adhesion molecules, which comprise a diverse set of proteins working as trans-synaptic anchors [18]. The actin cytoskeleton dynamics are orchestrated by the coordinated activity of actin-binding proteins [19,20], while cell adhesion molecules are subject to ectodomain shedding, a process that modifies pre- and postsynaptic contacts and mediates activity-dependent signaling [21,22]. Consistent with the main role of such pathways in regulating dendritic spine morphology and functions, here we investigated within DLPFC and hippocampus of SCZ brains, the expression levels of three synaptic elements that can be regarded as key spine shapers: the actin-binding protein cyclase-associated protein 2 (*CAP2*) [23], the sheddase a disintegrin and metalloproteinase 10 (*ADAM10*), and its binding partner the synapse-associated protein 97 (*SAP97*) [24], responsible for the trafficking of *ADAM10* and glutamate receptor subunits to the synaptic membrane [25]. In addition, we investigated whether the mRNA and protein expression of *CAP2*, *SAP97*, and *ADAM10* are also affected in patients with neurodegenerative disorders such as Parkinson’s Disease (PD) and Alzheimer’s Disease (AD), which are also characterized by spine defects.

## 2. Results

### 2.1. Colocalization of CAP2, SAP97, and ADAM10 in Primary Hippocampal Neurons

**Figure 1:**
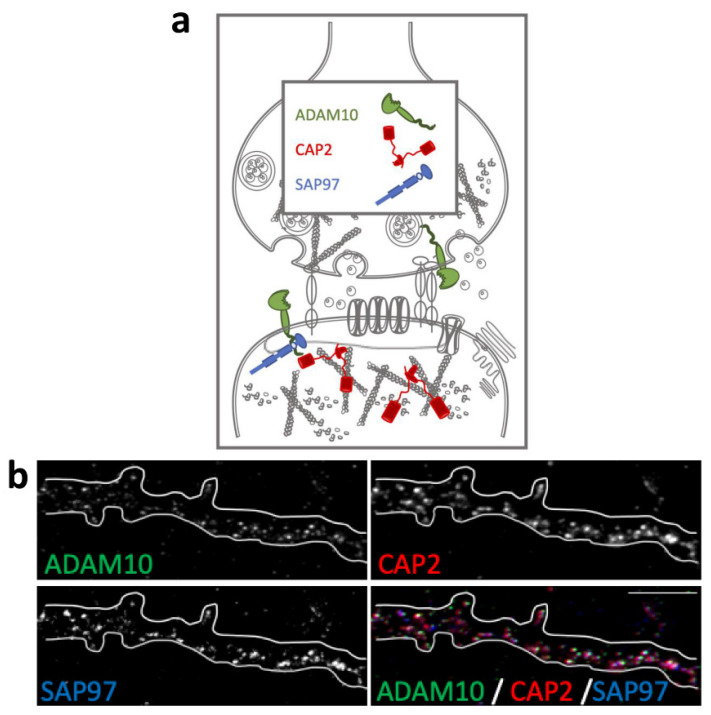
Colocalization of *CAP2*, *SAP97*, and *ADAM10* in primary hippocampal neurons. (**a**) Schematic representation of *CAP2*, *SAP97*, and *ADAM10* in the glutamatergic synapse. (**b**) Fluorescence immunocytochemistry of *ADAM10* (green), *CAP2* (red), and *SAP97* (blue) in primary hippocampal neurons. In the last panel (merge), a colocalization image is shown. Scale bar: 5 μm.

The actin-binding proteins *CAP2*, *ADAM10*, and its binding partner *SAP97* are synaptic proteins enriched in the postsynaptic compartment (Figure 1a) and their synaptic localization is finely modulated by activity-dependent synaptic plasticity [23,26]. To assess their colocalization in dendritic spines, we performed an immunocytochemistry experiment in primary hippocampal neuronal cultures. As shown in Figure 1b, the staining revealed that the spine shapers are localized along dendrites and in dendritic spines, where they colocalize in the head of the spines.

### 2.2. Correlation Analysis of CAP2, DLG1, and ADAM10 mRNA Expression with Age and PMI in the Post-Mortem Dorsolateral Prefrontal Cortex and Hippocampus of Schizophrenia Patients

Here we investigated the correlations between *CAP2*, *DLG1* (encoding *SAP97* protein), and *ADAM10* mRNA expression with demographic and post-mortem storage characteristics such as age and PMI, respectively (Table 1).

**Figure 2:**
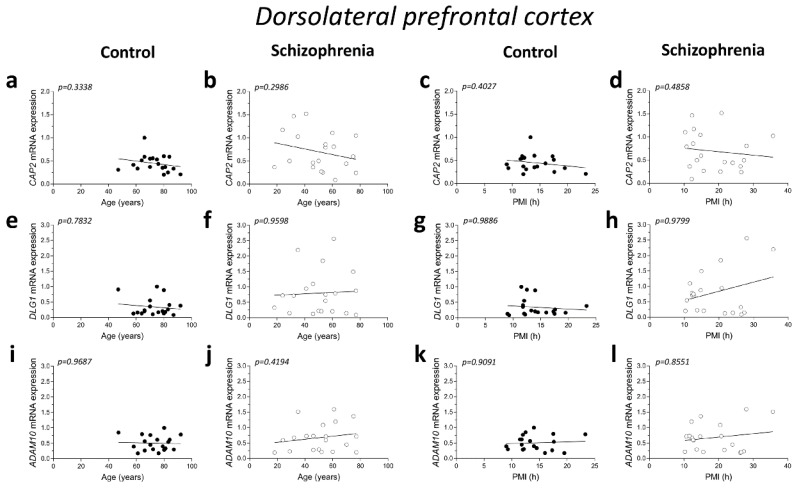

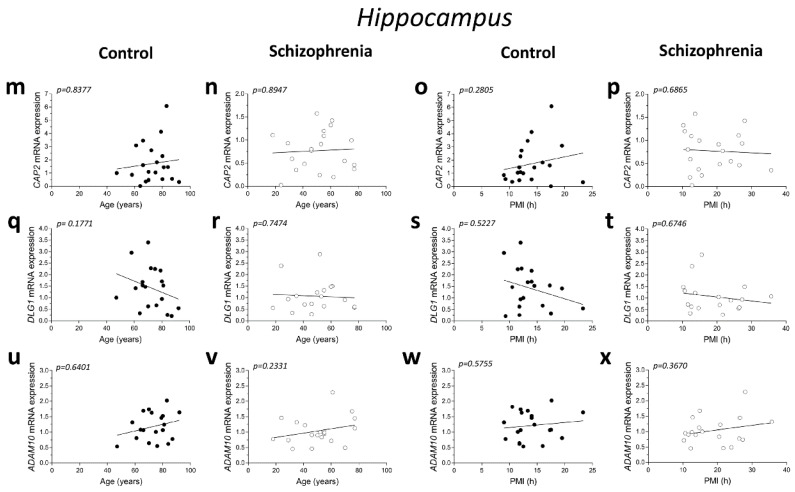
Correlation analysis of *CAP2*, *DLG1*, and *ADAM10* mRNA expression with age and PMI in the post-mortem dorsolateral prefrontal cortex and hippocampus of schizophrenia patients. Analysis of correlation between age and mRNA expression of *CAP2*, *DLG1*, and *ADAM10* in the dorsolateral prefrontal cortex of (**a**,**e**,**i**) control subjects (CTRL, *n* = 19) and (**b**,**f**,**j**) schizophrenia patients (SCZ, *n* = 20). Correlation analysis between PMI and mRNA expression of *CAP2*, *DLG1*, and *ADAM10* in the dorsolateral prefrontal cortex of (**c**,**g**,**k**) control subjects (*n* = 19) and (**d**,**h**,**l**) schizophrenia patients (*n* = 20). Analysis of correlation between age and mRNA expression of *CAP2*, *DLG1*, and *ADAM10* in the hippocampus of (**m**,**q**,**u**) control subjects (*CAP2*/*ADAM10*: *n* = 20; *DLG1*: *n* = 19) and (**n**,**r**,**v**) schizophrenia patients (*CAP2*: *n* = 20; *DLG1*: *n* = 18; *ADAM10*: *n* = 19). Correlation analysis between PMI and mRNA expression of *CAP2*, *DLG1*, and *ADAM10* in the hippocampus of (**o**,**s**,**w**) control subjects (*CAP2*/*ADAM10*: *n* = 20; *DLG1*: *n* = 19) and (**p**,**t**,**x**) schizophrenia patients (*CAP2*: *n* = 20; *DLG1*: *n* = 18; *ADAM10*: *n* = 19).

No significant correlations were found between mRNA expression and age in the DLPFC of both control and SCZ patients (*CAP2* CTRL: r = −0.2345; *p* = 0.3338; *n* = 19; *CAP2* SCZ: r = −0.2446; *p* = 0.2986; *n* = 20; *DLG1* CTRL: r = 0.06763; *p* = 0.7832; *n* = 19; *DLG1* SCZ: r = 0.01204; *p* = 0.9598; *n* = 20; *ADAM10* CTRL: r = 0.009662; *p* = 0.9687; *n* = 19; *ADAM10* SCZ: r = 0.1912; *p* = 0.4194; *n* = 20; Spearman correlation) (Figure 2a,b,e,f,i,j). Next, we investigated whether the mRNA levels correlated with PMI. Overall, qPCR results showed that *CAP2*, *DLG1*, and *ADAM10* transcript levels did not significantly change with PMI in the DLPFC of controls (*CAP2*: r = −0.2038; *p* = 0.4027; *n* = 19; *DLG1*: r = 0.003513; *p* = 0.9886; *n* = 19; *ADAM10*: r = −0.02811; *p* = 0.9091; *n* = 19; Spearman correlation) (Figure 2c,g,k) as well as in SCZ patients (*CAP2*: r = −0.1654; *p* = 0.4858; *n* = 20; *DLG1*: r = −0.006015; *p* = 0.9799; *n* = 20; *ADAM10*: r = −0.04361; *p* = 0.8551; *n* = 20; Spearman correlation) (Figure 2d,h,l).

Additionally, in the hippocampus we failed to find significant correlation between *CAP2*, *DLG1*, and *ADAM10* mRNA and age in SCZ patients and non-psychiatric controls (*CAP2* CTRL: r = 0.04893; *p* = 0.8377; *n* = 20; *CAP2* SCZ: r = 0.03161; *p* = 0.8947; *n* = 20; *DLG1* CTRL: r = −0.3232; *p* = 0.1771; *n* = 19; *DLG1* SCZ: r = 0.08165; *p* = 0.7474; *n* = 18; *ADAM10* CTRL: r = 0.1114; *p* = 0.6401; *n* = 20; *ADAM10* SCZ: r = 0.2872; *p* = 0.2331; *n* = 19; Spearman correlation) (Figure 2m,n,q,r,u,v). Moreover, we failed to observe any significant correlation between gene expression and PMI in the hippocampus of control individuals (*CAP2*: r = 0.2537; *p* = 0.2805; *n* = 20; *DLG1*: r = −0.1563; *p* = 0.5227; *n* = 19; *ADAM10*: r = 0.1332; *p* = 0.5755; *n* = 20; Spearman correlation) (Figure 2o,s,w) and in SCZ patients (*CAP2*: r = −0.09624; *p* = 0.6865; *n* = 20; *DLG1*: r = −0.1063; *p* = 0.6746; *n* = 18; *ADAM10*: r = 0.2193; *p* = 0.3670; *n* = 19; Spearman correlation) (Figure 2p,t,x).

### 2.3. Analysis of mRNA Expression of CAP2, DLG1, and ADAM10 in the Post-Mortem Dorsolateral Prefrontal Cortex and Hippocampus of SCZ Patients

**Figure 3:**
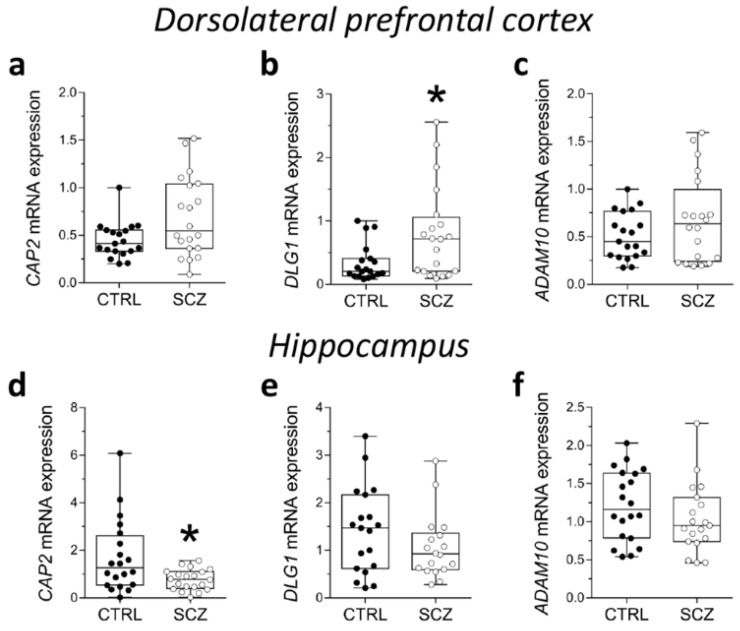
Analysis of mRNA expression of *CAP2*, *DLG1*, and *ADAM10* in the post-mortem dorsolateral prefrontal cortex and hippocampus of SCZ patients. Expression levels of (**a**) *CAP2*, (**b**) *DLG1* and (**c**) *ADAM10* mRNA in the post-mortem dorsolateral prefrontal cortex (DLPFC) of schizophrenia-affected patients (SCZ) (*n* = 20) and control subjects (CTRL) (*n* = 19). Expression levels of (**d**) *CAP2*, (**e**) *DLG1*, and (**f**) *ADAM10* mRNA in the post-mortem hippocampus of schizophrenic patients and controls (*CAP2*: CTRL/SCZ, *n* = 20; *DLG1*: CTRL, *n* = 19 SCZ, *n* = 18; *ADAM10*: CTRL, *n* = 20, SCZ, *n* = 19). *CAP2*, *DLG1*, and *ADAM10* transcript levels were detected by quantitative RT-PCR, normalized to the mean of two housekeeping genes (*ACTB* and *PPIA*), and expressed as 2^−ΔΔCt^. Each dot represents values from a single subject. * *p* < 0.05 compared to the control group (Mann–Whitney test).

Here, we evaluated potential alterations in *CAP2*, *DLG1*, and *ADAM10* mRNA expression in the DLPFC of SCZ patients compared to control subjects. Statistical analysis indicated no significant difference in *CAP2* and *ADAM10* transcript levels in SCZ patients, compared to non-psychiatric controls (*CAP2*: *p* = 0.0893, *ADAM10*: *p* = 0.4780; Mann–Whitney test) (Figure 3a,c). Conversely, we reported increased levels of *DLG1* transcript in SCZ patients compared to control subjects (*p* = 0.0407; Mann–Whitney test) (Figure 3b). Next, we extended gene expression analysis of *CAP2*, *DLG1*, and *ADAM10* to the post-mortem hippocampus of SCZ patients and control individuals. Interestingly, RT-PCR experiments revealed decreased *CAP2* mRNA levels in the SCZ group compared to control subjects (*p* = 0.0309; Mann–Whitney test) (Figure 3d). On the other hand, no significant difference in *DLG1* and *ADAM10* transcripts was observed between the two diagnostic groups (*DLG1*: *p* = 0.2485; *ADAM10*: *p* = 0.1682; Mann–Whitney test) (Figure 3e,f).

### 2.4. Correlation Analysis of CAP2, SAP97, and ADAM10 Protein Levels with Age and PMI in the Post-Mortem Dorsolateral Prefrontal Cortex and Hippocampus of Schizophrenia Patients

**Figure 4:**
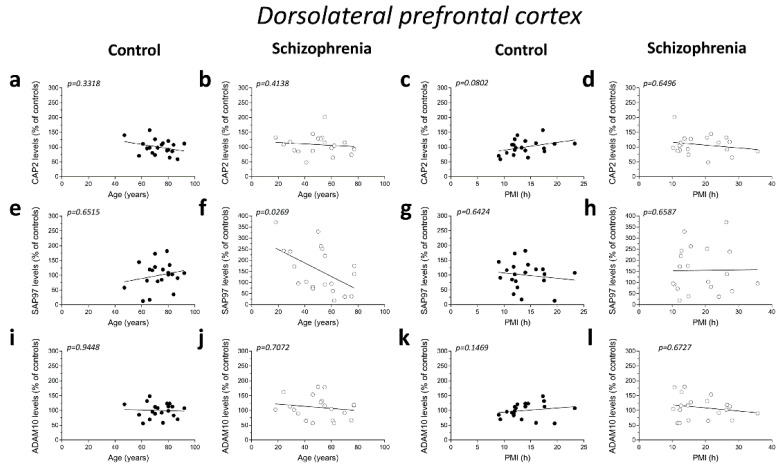

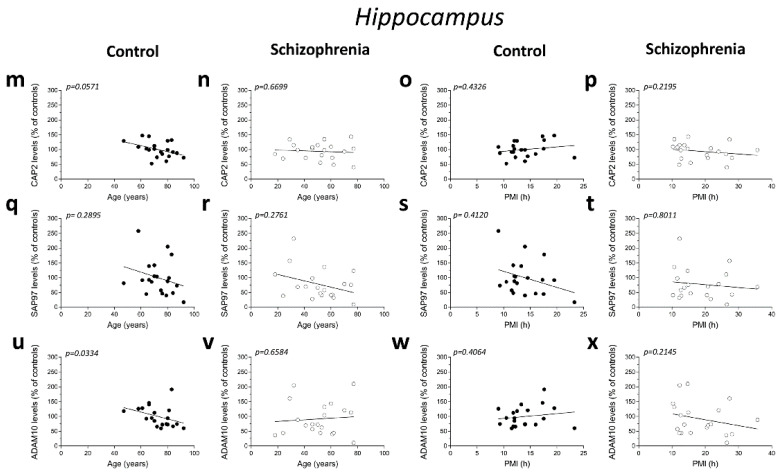
Correlation analysis of *CAP2*, *SAP97*, and *ADAM10* protein levels with age and PMI in the post-mortem dorsolateral prefrontal cortex and hippocampus of schizophrenia patients. Analysis of correlation between age and protein levels of *CAP2*, *SAP97*, and *ADAM10* in the dorsolateral prefrontal cortex of (**a**,**e**,**i**) control subjects (CTRL, *n* = 20) and (**b**,**f**,**j**) schizophrenia patients (SCZ, *n* = 20). Correlation analysis between PMI and protein levels of *CAP2*, *SAP97*, and *ADAM10* in the dorsolateral prefrontal cortex of (**c**,**g**,**k**) control subjects (*n* = 20) and (**d**,**h**,**l**) schizophrenia patients (*n* = 20). Analysis of correlation between age and protein levels of *CAP2*, *SAP97*, and *ADAM10* in the hippocampus of (**m**,**q**,**u**) control subjects (*n* = 20) and (**n**,**r**,**v**) schizophrenia patients (*n* = 20). Correlation analysis between PMI and protein levels of *CAP2*, *SAP97*, and *ADAM10* in the hippocampus of (**o**,**s**,**w**) control subjects (*n* = 20) and (**p**,**t**,**x**) schizophrenia patients (*n* = 20).

We analyzed possible age- and PMI-related variations in *CAP2*, *SAP97*, and *ADAM10* protein levels in the post-mortem DLPFC and hippocampus from individuals with SCZ and controls. Overall, we failed to observe any significant correlation in the DLPFC between the amount of proteins and the age in the non-psychiatric group (*CAP2*: r = −0.2288; *p* = 0.3318; *n* = 20; *SAP97*: r = 0.1076; *p* = 0.6515; *n* = 20; *ADAM10*: r = −0.01656; *p* = 0.9448; *n* = 20; Spearman correlation) (Figure 4a,e,i). Similarly, the results obtained in the SCZ patients did not show a significant correlation between *CAP2* and *ADAM10* levels and age (*CAP2*: r = −0.1935; *p* = 0.4138; *n* = 20; *ADAM10*: r = −0.08957; *p* = 0.7072; *n* = 20; Spearman correlation) (Figure 4b,j). Conversely, we reported a negative correlation between *SAP97* protein levels and age (r = −0.4938; *p* = 0.0269; *n* = 20; Spearman correlation) (Figure 4f). On the other hand, our results showed no correlation between *CAP2*, *SAP97*, and *ADAM10* protein expression and PMI in the DLPFC of both diagnostic groups (*CAP2* CTRL: r = 0.4005; *p* = 0.0802; *n* = 20; *CAP2* SCZ: r = −0.1083; *p* = 0.6496; *n* = 20; *SAP97* CTRL: r = −0.1107; *p* = 0.6424; *n* = 20; *SAP97* SCZ: r = 0.1053; *p* = 0.6587; *n* = 20; *ADAM10* CTRL: r = 0.3365; *p* = 0.1469; *n* = 20; *ADAM10* SCZ: r = −0.1008; *p* = 0.6726; *n* = 20; Spearman correlation) (Figure 4c,d,g,h,k,l). Finally, we analyzed the correlation of *CAP2*, *SAP97*, and *ADAM10* protein levels with age and PMI also in the hippocampus. In the control group, we did not observe significant correlation of *CAP2* (r = −0.4321; *p* = 0.0571; *n* = 20; Spearman correlation) (Figure 4m) and *SAP97* levels (r = −0.2492; *p*= 0.2895; *n* = 20; Spearman correlation) (Figure 4q) with the age, while we found a negative correlation between *ADAM10* and age (r = −0.4772; *p* = 0.0334; *n* = 20; Spearman correlation) (Figure 4u). Our results showed no significant correlation between *CAP2*, *SAP97*, and *ADAM10* protein levels and age in the SCZ patients (*CAP2*: r = −0.1016; *p* = 0.6699; *n* = 20; *SAP97*: r = −0.2559; *p* = 0.2761; *n* = 20; *ADAM10*: r = 0.1054; *p* = 0.6584; *n* = 20; Spearman correlation) (Figure 4n,r,v). Next, we analyzed the correlation between *CAP2*, *SAP97*, and *ADAM10* protein expression and PMI. We failed to find any significant correlation in both diagnostic groups (*CAP2* CTRL: r = 0.1859; *p* = 0.4326; *n* = 20; *CAP2* SCZ: r = −0.2872; *p* = 0.2195; *n* = 20; *SAP97* CTRL: r = −0.1942; *p* = 0.4120; *n* = 20; *SAP97* SCZ: r = −0.06015; *p* = 0.8011; *n* = 20; *ADAM10* CTRL: r = 0.1965; *p* = 0.4064; *n* = 20; *ADAM10* SCZ: r = −0.2902; *p* = 0.2145; *n* = 20; Spearman correlation) (Figure 4o,p,s,t,w,x).

### 2.5. Analysis of Protein Expression of CAP2, SAP97, and ADAM10 in the Post-Mortem Dorsolateral Prefrontal Cortex and Hippocampus of SCZ Patients

**Figure 5:**
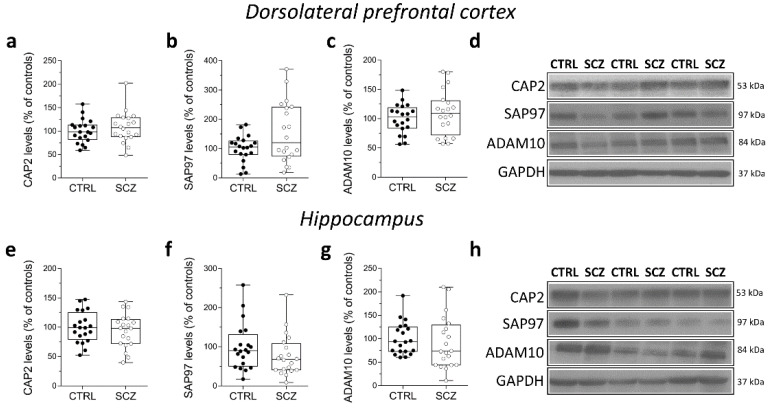
Analysis of protein expression of *CAP2*, *SAP97*, and *ADAM10* in the post-mortem dorsolateral prefrontal cortex and hippocampus of schizophrenia patients. Quantification of *CAP2*, *SAP97*, and *ADAM10* protein levels in the total homogenates of post-mortem (**a**–**c**) dorsolateral prefrontal cortex (DLPFC) and (**e**–**g**) hippocampus of schizophrenic subjects (SCZ, *n* = 20) and control group (CTRL, *n* = 20). The variations of *CAP2*, *SAP97*, and *ADAM10* levels in schizophrenic patients are expressed as a percentage (%) of the control subjects. All markers were normalized to GAPDH for variations in loading and transfer. (**d**,**h**) Representative images of immunoblots of *CAP2*, *SAP97*, *ADAM10* performed in the DLPFC and hippocampus of the control group and schizophrenia patients. Each dot represents values from a single subject. All experiments were analyzed by the Mann–Whitney test.

We also analyzed *CAP2*, *SAP97*, and *ADAM10* protein levels in total homogenates of DLPFC of the same cohort of post-mortem samples. Western blot analysis indicated no alterations of *CAP2*, *SAP97*, and *ADAM10* protein levels in the DLPFC of SCZ patients compared to non-psychiatric controls (*CAP2*: *p* = 0.4135; *SAP97*: *p* = 0.2110; *ADAM10* *p* = 0.4612; Mann–Whitney test) (Figure 5a–d). Similarly, in the hippocampus we found comparable *CAP2*, *SAP97*, and *ADAM10* protein levels between SCZ patients and control subjects (*CAP2*: *p* = 0.6205; *SAP97*: *p* = 0.1344; *ADAM10*: *p* = 0.2766; Mann–Whitney test) (Figure 5e–h).

### 2.6. Correlation Analysis of CAP2, SAP97, and ADAM10 mRNA and Protein Expression with Age and PMI in the Post-Mortem Superior Frontal Gyrus of Alzheimer’s and Parkinson’s Disease Patients

Synaptic dysfunction has been considered a major determinant of many neurological diseases, including AD, PD, and Huntington’s Disease [27,28]. Therefore, we extended our assessment of *CAP2*, *DLG1*, and *ADAM10* gene and protein expression levels to the post-mortem superior frontal gyrus (SFG) of patients affected by neurodegenerative diseases such as PD and AD.

First, we examined the correlations of *CAP2*, *DLG1*, and *ADAM10* mRNA with age and PMI in the SFG of AD and PD patients and the control group (Table 2).

**Figure 6:**
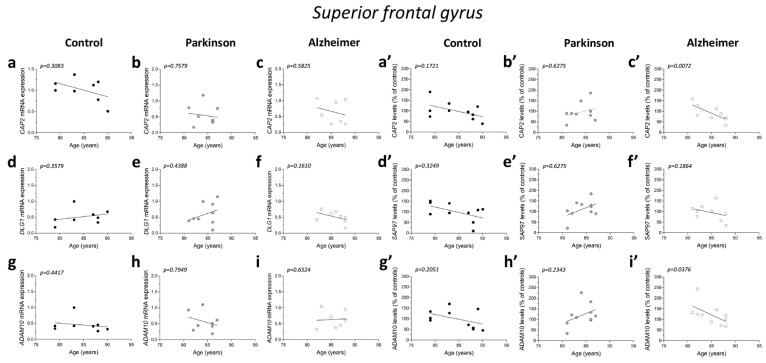

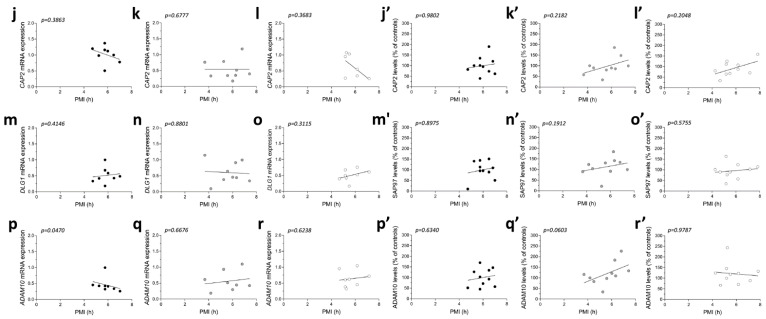
Correlation analysis of *CAP2*, *SAP97*, and *ADAM10* mRNA and protein expression with age and PMI in the post-mortem superior frontal gyrus of Alzheimer’s and Parkinson’s disease patients. Analysis of correlation between age and mRNA expression of *CAP2*, *DLG1*, and *ADAM10* in the superior frontal gyrus of (**a**,**d**,**g**) control subjects (CTRL, *n* = 8), (**b**,**e**,**h**) Parkinson’s disease patients (PD, *CAP2*/**DLG1* n* = 9, *ADAM10*: *n* = 8), and (**c**,**f**,**i**) Alzheimer’s (AD, *CAP2*: *n* = 7, *DLG1*/*ADAM10*: *n* = 8). Analysis of correlation between age and protein levels of *CAP2*, *SAP97*, and *ADAM10* in the superior frontal gyrus of (**a′**,**d′**,**g′**) control subjects (CTRL, *n* = 10), (**b′**,**e′**,**h′**) Parkinson’s disease patients (PD, *n* = 10), and (**c′**,**f′**,**i′**) Alzheimer’s disease patients (AD, *n* = 10). Analysis of correlation between PMI and mRNA expression of *CAP2*, *DLG1*, and *ADAM10* in the superior frontal gyrus of (**j**,**m**,**p**) control subjects (CTRL, *n* = 8), (**k**,**n**,**q**) Parkinson’s disease patients (PD, *CAP2*/**DLG1* n* = 9, *ADAM10*: *n* = 8), and (**l**,**o**,**r**) Alzheimer’s disease patients (AD, *CAP2*: *n* = 7, *DLG1*/*ADAM10*: *n* = 8). Analysis of correlation between PMI and protein levels of *CAP2*, *SAP97*, and *ADAM10* in the superior frontal gyrus of (**j′**,**m′**,**p′**) control subjects (CTRL, *n* = 10), (**k′**,**n′**,**q′**) Parkinson’s disease patients (PD, *n* = 10), and (**l′**,**o′**,**r′**) Alzheimer’s disease patients (AD, *n* = 10).

No significant correlations were observed between gene expression and age in the three diagnostic groups (*CAP2* CTRL: r = −0.4122; *p* = 0.3083; *n* = 8; *CAP2* PD: r = −0.1219; *p* = 0.7579; *n* = 9; *CAP2* AD: r = −0.2523; *p* = 0.5825; *n* = 7; *DLG1* CTRL: r = 0.3758; *p* = 0.3579; *n* = 8; *DLG1* PD: r = 0.2959; *p* = 0.4388; *n* = 9; *DLG1* AD: r = −0.5611; *p* = 0.1610; *n* = 8; *ADAM10* CTRL: r = −0.3152; *p* = 0.4417; *n* = 8; *ADAM10* PD: r = −0.122; *p* = 0.7949; *n* = 8; *ADAM10* AD: r = 0.1952; *p* = 0.6524; *n* = 8 Spearman correlation) (Figure 6a–i). We also failed to find any difference in the correlation of *CAP2*, *DLG1*, and *ADAM10* mRNA with PMI in AD and PD patients and control group (*CAP2* CTRL: r = −0.366; *p* = 0.3863; *n* = 8; *CAP2* PD: r = 0.1667; *p* = 0.6777; *n* = 9; *CAP2* AD: r = −0.4001; *p* = 0.3683; *n* = 7; *DLG1* CTRL: r = 0.3416; *p* = 0.4146; *n* = 8; *DLG1* PD: r = −0.06667; *p* = 0.8801; *n* = 9; *DLG1* AD: r = 0.4097; *p* = 0.3115; *n* = 8; *ADAM10* PD: r = 0.1812; *p* = 0.6676; *n* = 8; *ADAM10* AD: r = 0.2048; *p* = 0.6238; *n* = 8; Spearman correlation) (Figure 6j–o,q,r), except for *ADAM10* in the control group (r = −0.7319; *p* = 0.0470; *n* = 8; Spearman correlation) (Figure 6p).

Next, we investigated if the protein expression of these genes is correlated with both age and PMI in the same brain region. We found a significant negative correlation of *CAP2* and *ADAM10* protein levels with age in AD patients (*CAP2*: r = −0.8062; *p* = 0.0072; *n* = 10; *ADAM10*: r = −0.677; *p* = 0.0376; *n* = 10 Spearman correlation) (Figure 6c′,i′). No other significant correlation was found between our proteins of interest and age or PMI in the SFG of the three diagnostic groups (protein vs. age: *CAP2* CTRL: r =−0.4692; *p* = 0.1721; *n* = 10; *CAP2* PD: r = 0.1757; *p* = 0.6275; *n* = 10; *SAP97* CTRL: r = −0.3457; *p* = 0.3249; *n* = 10; *SAP97* PD: r = 0.1757; *p* = 0.6275; *n* = 10; *SAP97* AD: r = −0.4554; *p* = 0.1864; *n* = 10; *ADAM10* CTRL: r = −0.4383 *p* = 0.2051; *n* = 10; *ADAM10* PD: r = 0.414; *p* = 0.2343; *n* = 10; Spearman correlation) (Figure 6a′,b′,d′–h′) (protein vs. PMI: *CAP2* CTRL: r = 0.01231; *p* = 0.9802; *n* = 10; *CAP2* PD: r = 0.4303; *p* = 0.2182; *n* = 10; *CAP2* AD: r = 0.439; *p* = 0.2048; *n* = 10; *SAP97* CTRL: r = −0.04924; *p* = 0.8975; *n* = 10; *SAP97* PD: r = 0.4545.; *p* = 0.1912; *n* = 10; *SAP97* AD: r = 0.2012; *p* = 0.5755; *n* = 10; *ADAM10* CTRL: r = 0.1723; *p* = 0.6340; *n* = 10; *ADAM10* PD: r = 0.6242; *p* = 0.0603; *n* = 10; *ADAM10* AD: r = −0.0122; *p* = 0.9787; *n* = 10; Spearman correlation) (Figure 6j′–r′).

### 2.7. Analysis of CAP2, DLG1, and ADAM10 Transcript Levels in the Post-Mortem Superior Frontal Gyrus of Alzheimer’s and Parkinson’s Disease Patients

**Figure 7:**
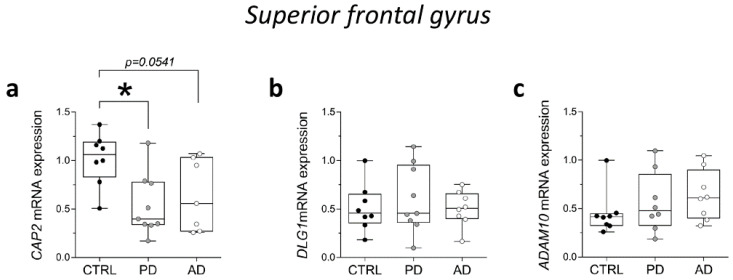
Analysis of transcript of *CAP2*, *DLG1*, and *ADAM10* in the post-mortem superior frontal gyrus of Alzheimer’s and Parkinson’s disease patients. mRNA expression levels of (**a**) *CAP2* (CTRL, *n* = 8, PD, *n* = 9, AD, *n* = 7), (**b**) *DLG1* (CTRL, *n* = 8, PD, *n* = 9, AD, *n* = 8), and (**c**) *ADAM10* (CTRL/PD/AD, *n* = 8) in the post-mortem superior frontal gyrus. *CAP2*, *DLG1*, and *ADAM10* transcript levels were detected by quantitative RT-PCR, normalized to the mean of two housekeeping genes (ACTB and PPIA), and expressed as 2^−ΔΔCt^. Each dot represents values from a single subject. * *p* < 0.05 compared to the control group (Mann–Whitney test).

Then, we analyzed *CAP2*, *DLG1*, and *ADAM10* gene expression levels in the post-mortem SFG of PD and AD. RT-PCR analysis showed a significant reduction of *CAP2* transcript in PD patients and a decreasing trend in AD patients, compared with non-neurological subjects (CTRL vs. PD *p* = 0.0111; CTRL vs. AD *p* = 0.0541; Mann–Whitney test) (Figure 7a). Conversely, we did not observe any alteration in *DLG1* and *ADAM10* mRNA expression levels between groups (*DLG1*: CTRL vs. PD *p* = 0.7430; CTRL vs. AD *p* = 0.8785; *ADAM10*: CTRL vs. PD *p* = 0.3823; CTRL vs. AD *p* = 0.1304; Mann–Whitney test) (Figure 7b,c).

### 2.8. Analysis of Protein Expression of CAP2, SAP97, and ADAM10 in the Post-Mortem Superior Frontal Gyrus of Alzheimer’s and Parkinson’s Disease Patients

**Figure 8:**
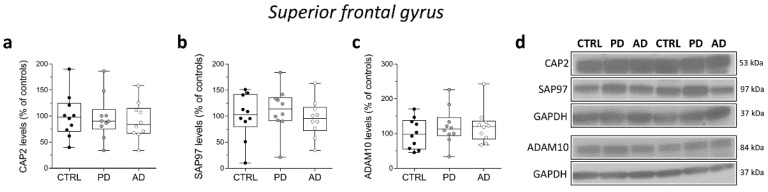
Analysis of protein expression of *CAP2*, *SAP97*, and *ADAM10* in the post-mortem superior frontal gyrus of Alzheimer’s and Parkinson’s disease patients. Quantification of western blot analysis of (**a**) *CAP2*, (**b**) *SAP97*, and (**c**) *ADAM10* protein levels in the total homogenates of post-mortem superior frontal gyrus (SFG) of Parkinson’s (*n* = 10), Alzheimer’s disease patients (*n* = 10), and control subjects (*n* = 10). The variations of *CAP2*, *SAP97*, and *ADAM10* levels in patients with Parkinson’s and Alzheimer’s disease are expressed as a percentage (%) of the control subjects. All markers were normalized to GAPDH for variations in loading and transfer. (**d**) Representative images of immunoblots of *CAP2*, *SAP97*, and *ADAM10* performed in the SFG of Parkinson’s, Alzheimer’s disease patients, and control subjects. Each dot represents values from a single subject. All experiments were analyzed by the Mann–Whitney test.

Finally, in the same brain samples, we performed Western blot analysis to investigate eventual variations in *CAP2*, *SAP97*, and *ADAM10* protein content in individuals with PD and AD. Our analyses showed unaltered *CAP2*, *SAP97*, and *ADAM10* protein levels in the SFG of PD and AD patients compared to control subjects (*CAP2*: CTRL vs. PD *p* = 0.6842; CTRL vs. AD *p* = 0.6842; *SAP97*: CTRL vs. PD *p* = 0.6842; CTRL vs. AD *p* = 0.6305; *ADAM10*: CTRL vs. PD *p* = 0.5288; CTRL vs. AD *p* = 0.5787; Mann–Whitney test) (Figure 8a–d).

## 3. Discussion

Several psychiatric and neurological disorders are associated with alterations in spine number and dendritic arborization [29,30]. The spine shapers *CAP2*, *SAP97*, and *ADAM10* are localized in the postsynaptic compartment and their localization is under the control of activity-dependent synaptic plasticity [23,26]. *CAP2* translocation into spines is required for spine enlargement upon long-term potentiation induction [23]. *SAP97* controls the abundance of *ADAM10* at the synapse and their association is essential for the long-term depression-triggered spine shrinkage [26]. Based on the well-recognized synaptic defects in the SCZ brain [12], here we investigated the mRNA and protein levels of *CAP2*, *SAP97*, and *ADAM10* in the post-mortem brain of SCZ patients. In particular, we performed the analysis in total homogenates of DLPFC and hippocampus, two brain regions involved in deregulated cortical–subcortical network typical of this psychiatric disorder [31,32,33,34].

Our data indicated a significant increase of *DLG1* mRNA levels within DLPFC, but not in the hippocampus of SCZ patients, when compared to controls. Conversely, no changes were observed in the expression levels of the protein encoded by the *DLG1* gene, *SAP97*, in both brain regions.

Taken together, these results are puzzling since it has been demonstrated a pivotal role of this synaptic element in regulating the trafficking of the glutamate AMPA receptors, whose expression, and function have been reported to be altered in SCZ [35]. In this regard, compelling evidence, including genetic-imaging studies, suggests that genetic variants of the *DLG1* gene are functionally linked to abnormal cognitive patterns in SCZ patients [36,37,38,39,40]. In particular, Xu and colleagues reported that *SAP97* rs3915512 polymorphism in patients with first-episode schizophrenia was associated with low structural and functional connectivity within the orbitofrontal–striatal–thalamic circuitry [41]. Despite this finding, protein expression analysis of *SAP97* in DLPFC reported conflicting data, showing either a reduction [42] or increased protein amount in SCZ brains, compared to healthy controls [43]. In addition, no difference was detected in *DLG1* gene expression in both DLPFC and occipital cortex of post-mortem old adults with SCZ [44].

Notably, we observed a decrease of *CAP2* transcript level, selectively in the hippocampus, but not in the DLPFC of SCZ patients, while no changes in *CAP2* protein levels were detected in both brain regions. To the best of our knowledge, this is the first work reporting *CAP2* mRNA reduction in the post-mortem brain of SCZ patients. In humans, the *CAP2* gene is located on chromosome 6p22.3. Interestingly, patients with an interstitial 6p22−24 deletion syndrome show a complex and variable phenotype including a developmental delay and cognitive disorders [45,46]. It is worth mentioning that the expression of CAP1, another member of the CAP family, is increased in the mediodorsal thalamus of SCZ patients compared to control subjects [47] and is altered in the cortex of an animal model of SCZ [48]. Despite *CAP2* transcript alteration in the hippocampus of SCZ patients, we found that the protein levels were unaffected in this brain area thus making it difficult to argue a direct consequence of mRNA reduction in modulating excitatory synaptic transmission. Further investigations are warranted to understand the relevance of *CAP2* at the glutamatergic synapse of the SCZ brain.

Overall, our data indicate alterations in both *DLG1* (increase) and *CAP2* (decrease) gene expression in SCZ brains but not in their corresponding protein levels. It should be remarked that our analysis has been carried out using total homogenate extracts. Therefore, future studies are needed to assess whether differences in the expression levels of these proteins occur in selective neuronal compartments, like the postsynaptic fraction where they exert their specific function.

Our data also indicated an unaltered mRNA and protein expression of *ADAM10* in the post-mortem brain of SCZ patients, compared to normal controls in both DLPFC and hippocampus. These results are in line with a recent report indicating no changes in transcript and protein expression of *ADAM10* in post-mortem PFC of SCZ patients [49]. However, a very recent study showed a significant reduction of *ADAM10* mRNA in several brain regions of SCZ patients [50]. The apparent discrepancy between our results on *CAP2*, *SAP97*, and *ADAM10* mRNA and protein expression and those obtained from other cohorts of post-mortem SCZ brains should take into account diverse confounding variables, including antipsychotics, age, disease duration, and gender. In particular, pharmacological therapy could have played a role in regulating the expression of these synaptic proteins at multiple levels, such as type of antipsychotic, dosage, and duration of treatment. Consistent with this assumption, evidence suggests that the expression of key scaffold proteins can be significantly affected in animal models exposed to antipsychotic drugs [51,52,53]. Moreover, we found that the age of SCZ patients was significantly lower than control individuals. This is in line with literature reporting a reduced life expectancy in patients with SCZ compared with the general population [54,55]. On the other hand, we did not find any effect of gender, so that, therefore, should not have affected our results.

In the present work, as a reference of synaptopathy-related diseases, we analyzed *CAP2*, *SAP97*, and *ADAM10* expression in post-mortem brains of patients affected by PD and AD. Indeed, aberrant synapse functioning is a common trait of several brain disorders, including the aforementioned neurodegenerative diseases [27]. Moreover, the relevance of postsynaptic proteins in spine pathology is also supported by evidence showing alterations of spine shapers in AD [23,56]. Consistent with this, *CAP2* protein levels were found to be reduced in the hippocampus of patients and animal models of AD [23]. It is noteworthy that even though our results show a remarkable decreasing tendency of *CAP2* mRNA levels in the SFG of AD individuals, *CAP2* protein levels were not affected, confirming the results obtained in previous studies [23]. *CAP2* has never been analyzed in PD, while *ADAM10* and *SAP97* gene variants have already been associated with PD [57,58]. In the present work, we show a significant reduction of *CAP2* mRNA levels in the SFG of the PD group, while no alterations in *SAP97* and *ADAM10* expression were detected in patients, compared to controls. In contrast to our observations, earlier immunohistochemical studies carried out in the human post-mortem hippocampus of PD patients showed a significant increase of *SAP97* [59]. This synapse-associated protein was also altered in the striatum of animal models of PD and Levodopa-induced dyskinesia [60]. We can hypothesize that the alterations in *SAP97* expression in PD can depend on different factors, including the brain area analyzed, the disease stage, and drug administration. In conclusion, our data suggest that aberrant gene expression of spine shapers may contribute significantly to synaptic dysfunction-related neuropsychiatric disorders. The involvement of key molecules of the synapse may highlight the need for a more vigorous translational approach towards the search of novel molecular targets paving the way for innovative treatments beyond or in augmentation with the ones presently available.

## 4. Materials and Methods

### 4.1. Neuronal Cultures Preparation and Immunocytochemistry

Hippocampal neuronal primary cultures were prepared from embryonic day 19 (E19) rat hippocampi as previously described [61]. For colocalization studies, day in vitro (DIV) 14 hippocampal neurons were fixed with 4% Paraformaldehyde (PFA)—4% sucrose in PBS solution for 5 min at 4 °C and washed several times with PBS. Cells were permeabilized with 0.1% Triton X-100 in PBS for 15 min at room temperature (RT) and then blocked with 5% bovine serum albumin (BSA) in PBS for 1 h at RT. Cells were then labeled with primary antibodies at 4 °C overnight. The following antibodies were used: anti-*CAP2* (anta Cruz Biotechnology, cod. SC-167378), anti-*SAP97* (Enzo, cod. ADI-VAM-PS005), and anti-*ADAM10* (Abcam, cod. ab39153). Cells were washed and then incubated with secondary antibodies for 1 h at RT. The Alexa Fluor dye secondary antibodies used (donkey anti-rabbit-Alexa488, donkey anti-mouse-Alexa555, donkey anti-goat-Alexa647) were purchased from Thermo Fisher Scientific. After, the cells were washed in PBS and mounted on glass slides with Fluoromount mounting medium (Sigma Aldrich 20149,Milano, Italy). Images for the analysis of neuronal spine morphology were acquired with an Airyscan (resolution 100–120 nm) microscopy using Zeiss LSM 900, 63× oil objective, PLAN Apochromat, NA 1.42.

### 4.2. Human Post-Mortem Tissue Collection

Human tissue collection DLPFC and hippocampus samples from post-mortem brains of non-psychiatric controls and SCZ patients (*n* = 20/brain region/clinical condition) were obtained from The Human Brain and Spinal Fluid Resource Center (Los Angeles Healthcare Center, Los Angeles, CA, USA). Clinical diagnosis of SCZ was performed according to DSMIII-R criteria. Demographic characteristics of control and SCZ subjects are described in Table 1 and Table S1. We obtained human SFG samples of normal controls, PD, and AD patients from The Netherlands Brain Bank (Netherlands Institute for Neuroscience, Amsterdam, open access: www.brainbank.nl, accessed on 27 January 2022). We selected cases with a clinical diagnosis of AD (*n* = 10) and neuropathological staging of Braak ≥ 5. PD patients (*n* = 10) were characterized by Braak LB stage ≥ 4. Controls (*n*  =  10) were adults without cognitive decline and Braak ≤ 3 in accordance with the Braak and Braak criteria [62]. The control subjects had no known clinical history of neurological or psychiatric disorders and were also fully neuropathologically evaluated to confirm that they were free of neurodegenerative pathologies. AD patients had a clinical diagnosis of dementia or probable AD, according to the National Institute of Neurological and Communicative Disorders and Stroke and the Alzheimer’s Disease and Related Disorders Association (NINCDS-ADRDA) criteria [63]. Clinical diagnosis of PD was based on diagnostic procedure according to the UK Brain Bank criteria for PD [64] and confirmed by neuropathological findings [65]. Frozen tissues were pulverized in liquid nitrogen and stored at −80 °C for subsequent processing.

### 4.3. RNA Extraction and Quantitative RT-PCR Analysis

Total RNA was extracted from post-mortem tissues using RNeasy^®^ mini kit (Qiagen, Hilden, Germany) according to the manufacturer’s instructions (Querques et al. 2015). Total RNA was purified to eliminate potentially contaminating genomic DNA using recombinant DNase (Qiagen, Hilden, Germany). RNA integrity number (RIN) of samples was assessed using Agilent 2100 Bioanalyzer Expert (Santa Clara, CA, USA) and BioRad Experion Automated electrophoresis Station (Hercules, CA, USA) prior to cDNA synthesis using Transcriptor First Strand cDNA Synthesis kit (Roche Diagnostics, Mannheim, Germany). A total of 1 μg of total RNA of each sample was reverse transcribed with QuantiTect Reverse Transcription (Qiagen, Hilden, Germany) using oligo-dT and random primers according to the manufacturer’s instructions. Quantitative RT-PCR with Real Time ready catalogue Assays (Roche Diagnostics) and LightCycler^®^ 480 Probe Master (Roche Diagnostics) was performed on a Light Cycler 480 Real Time PCR thermocycler with 96-well format (Roche Diagnostics). All measurements from each subject were performed in duplicate. *CAP2*, *DLG1*, and *ADAM10* mRNA expression levels were normalized to the mean of two housekeeping genes: β-actin (*ACTB*) and cyclophilin (*PPIA*). The following protocol was used: 10 s for initial denaturation at 95 °C followed by 40 cycles consisting of 10 s at 94 °C for denaturation, 10 s at 60 °C for annealing, and 6 s for elongation at 72 °C temperature. The following primers were used for *CAP2*, *DLG1*, and *ADAM10* cDNA amplification: *CAP2* forward, 5′-GCC GCC TGG AGT CGC TGT C-3′ and *CAP2* reverse, 5′-AAA ACT CGG CCA CCA TAC TGT CCA-3′; *DLG1* forward, 5′-GAG ATG ACT CAA GTA TTT TCA TTA CCA-3′ and *DLG1* reverse, 5′-CAC GAA CAT CTA CTT CAT TTA CTC G-3′; *ADAM10* forward, 5′-CTGCCCAGCATCTGACCCTAA-3′, and *ADAM10* reverse, 5′-TTG CCA TCA GAA CTG GCA CAC-3′. mRNA expression was calculated using the geometric mean of the two reference genes selected and the relative quantification method (2^−ΔΔCt^).

### 4.4. Western Blotting

Frozen, powdered samples from post-mortem DLPFC and hippocampus and from SFG of respective brain banks were sonicated in 1% SDS and boiled for 10 min. Aliquots (2 µL) of the homogenate were used for protein determination using a BioRad Protein Assay kit. Equal amounts of total proteins (30 µg) for each sample were loaded on precast 4–20% gradient gels (BioRad Laboratories, Hercules, CA, USA). Proteins were separated by SDS-PAGE and transferred to PVDF membranes (GE Healthcare, Chicago, IL, USA) via the Trans Blot Turbo System (BioRad Laboratories, Hercules, CA, USA). To investigate the targets of interest the blots were incubated with antibodies against *CAP2* (1:1000; 15865-1-AP, Proteintech), *SAP97* (1:1000; ADI-VAM-PS005, Enzo Life Sciences), and *ADAM10* (1:4000; AbCaM Ab 39153). GAPDH (1:1000; sc-32233, Santa Cruz Biotechnology) was used to normalize the levels of analyzed proteins for variations in loading and transfer. All blots were incubated in horseradish peroxidase-conjugated secondary antibodies and target proteins visualized by ECL detection (Pierce, Rockford, IL, USA), followed by quantification through the “Quantity One” software (BioRad Laboratories, Hercules, CA, USA). Normalized values were then averaged and used for statistical comparisons. All representative blots shown in the figures arise from cut-out and pasted bands for reassembling the image. Of note, for each graph, the representative bands come from the same films.

### 4.5. Statistical Analysis

Normal distribution assumption for continuous variables was checked by Shapiro–Wilks and Kolmogorov–Smirnov tests. We observed a non-normal distribution of our data; therefore, a nonparametric approach was used for all statistical analyses. Data are reported as medians, along with interquartile range (first-third quartiles—IQR). Statistical analysis of qPCR and western blot experiments was performed in GraphPad Prism 7 by Mann–Whitney test. Statistical significance was also corrected for multiple comparisons using the Bonferroni-Dunn method (see Table S2). Spearman’s nonparametric correlation was used to test possible associations between nonparametric variables. Asterisks denote statistical significance as calculated by the specific statistical tests (*, *p* < 0.05).

## 5. Conclusions

In conclusion, we reported increased expression of *DLG1* transcript in DLPFC and a reduction in *CAP2* mRNA expression in the hippocampus of post-mortem SCZ brains, thus suggesting an overall altered expression of the genes encoding these dendritic spine proteins in SCZ.

## Figures and Tables

**Table 1:** Demographic and clinical characteristics of control subjects and schizophrenia patients.

Characteristics	Control	Schizophrenia	*p*-Value
Subjects (total number)	20	20	--
Gender (M/F)	16/4	12/8	0.301 ^a^
Age (years, median (IQR))	73.50 (66.00–80.25)	52.50 (39.50–61.25)	<0.001 ^b^
PMI (hours, median (IQR))	12.90 (11.80–16.32)	15.25 (12.52–24.58)	0.020 ^c^
pH (median, (IQR))	6.54 (6.49–6.63)	6.50 (6.42–6.56)	0.485 ^c^

Abbreviations: M/F: number of males/females; PMI: post-mortem interval; IQR: Interquartile Range (i.e., first-third quartiles). Continuous variables are reported as median along with IQR; ^a^ Chi-Square test; ^b^ two-sample *t*-test; ^c^ two-sample *t*-test on log-transformed values.

**Table 2:** Demographic and clinical characteristics of control subjects, Alzheimer’s and Parkinson’s disease patients.

Characteristics	Control		Alzheimer’s Disease		Parkinson’s Disease	
	N	Median	N	Median	N	Median
*Subjects (total number)*	10		10			10
*Gender (M/F)*	10/0		10/0		10/0	
*Age (years, median (IQR))*		85.00 (79.00–88.25)		85.50 (82.00–88.00)		85.00 (81.75–86.00)
*PMI (hours, median (IQR))*		5.87 (5.62–6.58)		5.37 (5.04–6.42)		5.79 (4.42–6.45)
*Amyloid (A/B/C/O)*	6/2/0/2		0/1/9/0		3/3/0/4	
*Braak (0/I/II/III/IV/V/VI)*	0/0/3/5/2/0/0		0/0/0/0/0/7/3		0/0/3/4/0/0/0	
*Braak LB (0/I/II/III/IV/V/VI)*	2/2/0/0/0/0/0		0/0/0/0/0/0/0		0/0/0/0/2/2/6	
*Liquor pH (median (IQR))*		6.37 (6.29–6.72)		6.28 (6.13–6.49)		6.33 (6.16–6.72)

Abbreviations: M/F: males/females; PMI: post-mortem interval; IQR: interquartile range (i.e., first-third quartiles); N: number. Continuous variables are reported as median along with IQR.

## Data Availability

The data that support the findings of this study are available from the corresponding author upon reasonable request.

## Supplementary Materials

The following supporting information can be downloaded at: https://www.mdpi.com/article/10.3390/ijms23031539/s1.
