# Supplementary material for: Analysis of mRNA and Protein Levels of CAP2, DLG1 and ADAM10 Genes in Post-Mortem Brain of Schizophrenia, Parkinson’s and Alzheimer’s Disease Patients
Source: Int J Mol Sci. 2022 Jan 28;23(3):1539. doi: 10.3390/ijms23031539 (PMC8835961; doi:10.3390/ijms23031539)
Supplement: Supplementary file 1 [file ijms-23-01539-s001.zip › ijms-1513888-supplementary.pdf]

Supplemental Information for

Analysis of mRNA and protein levels of CAP2, DLG1 and ADAM10 genes in post-mortem brain of schizophrenia, Parkinson's and Alzheimer's disease patients

Anna Di Maio[1,2,#], Arianna De Rosa[1#], Silvia Pelucchi[3], Martina Garofalo[1,4] , Benedetta Marciano[1], Tommaso Nuzzo[1,4], Fabrizio Gardoni[3], Andrea M. Isidori[2], Monica Di Luca[3], Francesco Errico[1,5,], Andrea De Bartolomeis[6], Elena Marcello[3,*] and Alessandro Usiello[1,4,*]

[1] Laboratory of Translational Neuroscience, CEINGE Biotecnologie Avanzate, 80145, Naples, Italy, dimaio@ceinge.unina.it (A.D.M.); derosaar@ceinge.unina.it (A.D.R.); garofalom@ceinge.unina.it (M.G.); marcianob@ceinge.unina.it (B.M.); nuzzo@ceinge.unina.it (T.N.);

[2] Department of Experimental Medicine, Sapienza University of Rome, 00185, Rome, Italy; andrea.isidori@uniroma1.it (A.M.I.);

[3] Department of Pharmacological and Biomolecular Sciences (DiSFeB), University of Milan, 20133, Milan, Italy; silviapelucchi87@gmail.com (S.P.); fabrizio.gardoni@unimi.it (F.G.); monica.diluca@unimi.it (M.D.L.) elena.marcello@unimi.it (E.M.);

[4] Department of Environmental, Biological and Pharmaceutical Science and Technologies, Università degli Studi della Campania "Luigi Vanvitelli", 81100, Caserta, Italy; usiello@ceinge.unina.it (A.U.);

[5] Department of Agricultural Sciences, University of Naples "Federico II", Naples, Italy; francesco.errico@ unina.it (F.E.);

[6] Section of Psychiatry Laboratory of Molecular and Translational Psychiatry, Department of Neuroscience, Reproductive Science and Odontostomatology School of Medicine, University "Federico II", Naples, Italy; adebarto@unina.it (A.D.B.)

[#] Share the Co-First authorship

[*] usiello@ceinge.unina.it; Tel.: +39-0813737899 (A.U.); elena.marcello@unimi.it; Tel.: +39 02 50318314 (E.M.).

Table S1. Demographic characteristics, comorbidities, clinical diagnosis of each control subject and schizophrenia patient.

Control						Schizophrenia						
ID	*Sex*	*Age (years)*	*PMI (hours)*	*pH*	*Clinical Diagnosis*	*ID*	*Sex*	*Age (years)*	*PMI (hours)*	*pH*	*Clinical Diagnosis*	*Antipsychotic medication*
1	M	47	12.5	6.53	CA (esophagus) with metastases to the liver	*21*	M	46	21.7	6.45	Schizophrenia, Depression, Bipolar, Seizure disorder, Epilepsy, Substance abuse	Quietapine
2	M	66	17.3	6.49	CA (lung), COPD	*22*	M	55	10.7	N.A.	Schizophrenia, Suicide, Overdose, Depression, Anxiety, Hypochondriasis	Risperidone, Fluphenazine
3	F	92	23.3	6.75	CA (uterus, stomach), Congestive hearth failure, Hypertension, Macular degeneration,	*23*	M	53	20.5	N.A.	Schizophrenia, Depression, Bipolar, CA (lung) Paranoia, Psychosis, Hypertension, Anxiety	Olanzapine, Fluphenazine
4	M	84	11.8	6.79	CA (stomach), Renal failure, acute, COPD	*24*	M	70	24.0	6.56	Schizophrenia, Paranoia, Aggressive behavior, Dementia, Impulse disorder, Tuberculosis	N.A.
5	M	70	11.8	6.62	Coronary hearth disease, Leukemia, Type I diabetes, Myocardial infarction, Congestive	*25*	M	35	35.7	6.51	Schizophrenia, Alcohol abuse	Risperidone, Haloperidol
6	M	87	9.3	6.76	Congestive hearth failure, Atherosclerosis, COPD	*26*	F	32	12.3	6.51	Schizophrenia, Alcohol abuse history	N.A.
7	M	58	9.0	6.32	CA (colon)	*27*	M	61	28.0	6.73	Schizophrenia, Aggressive behavior, Suicide, Attempts, Anxiety, Tobacco abuse, Asthma	Thioridazine, Fluphenazine, Quietapine, Paliperidone
8	M	68	10.5	N.A.	CA (lung), Alcohol abuse, Type I diabetes, Transient Ischemic Attack	*28*	F	41	20.8	6.39	Schizophrenia, Suicide, Stabbing, Psychosis, Disassociated Disorders, Electroconvulsive	Quietapine, Haloperidol, Risperidone
9	M	80	14.0	6.49	CA (bladder), Hypertension, Diabetes type II, CVA	*29*	F	50	13.7	N.A.	Schizophrenia, Suicide, Overdose, Aggressive behavior, Depression, Migraine, Hallucination	Risperidone
10	M	76	16.0	6.55	CA (lung). Pulmonary emphysema	*30*	M	18	26.3	6.72	Schizophrenia, Mentally retarded (clinical only), Attention Deficit Disorder, Sleep Apnea	Risperidone
11	M	75	11.5	6.6	CA (prostate) Coronary hearth disease, Hypertension	*31*	M	46	11.6	6.41	Schizophrenia, Suicide, hanging, Alcohol abuse, Substance abuse (not Alcohol), Depression	Quietapine, Risperidone
12	M	66	13.3	N.A.	CA (larynx) Metastasis to bone and liver, Type I diabetes	*32*	F	29	27.3	6.4	Schizophrenia, Depression, Seizure Disorder, Attention Deficit Disorder, Aggressive behavior	Aripiprazole, Risperidone
13	M	64	17.5	6.63	Lymphoma, Coronary Artery Disease	*33*	M	60	10.3	N.A.	Schizophrenia, Depression, Bipolar, Alcohol abuse, Psychotic disorder, Inappropriate sexual	Ziprasidone, Risperidone
14	M	80	12.0	N.A.	CA (kidney) Hypertension, Atrial fibrillation, Macular degeneration, COPD	*34*	F	75	14.9	N.A.	Schizophrenia, CA (pancreas), Dementia, Therapeutic lobotomy, Dysphagia	Risperidone
15	F	83	17.6	6.41	CA (breast, uterus, colon), Macular degeneration, Chronic urinary tract infection	*35*	F	77	14.7	6.38	Schizophrenia, Alcohol abuse, Depression, Bipolar and Seizure disorder	Fluphenazine, Risperidone
16	F	79	14.0	N.A.	Coronary hearth disease, Hypertension	*36*	M	77	26.5	6.56	Schizophrenia, Hypertension, Dementia, COPD	Quietapine
17	M	61	19.5	6.29	Normal	*37*	M	24	12.8	6.47	Schizophrenia, Depression, Substance abuse (not Alcohol), Suicide Attempts, Alcohol abuse	Aripiprazole, Quietapine
18	M	70	12.0	N.A.	Renal failure, acute, Diabetes type I	*38*	F	62	12.2	6.67	Schizophrenia, Stroke/CVA, Depression, Hypertension, Diabetes type I	Quietapine
19	M	72	12.2	6.54	COPD, Pulmonary emphysema	*39*	F	52	15.6	6.49	Schizophrenia, Depression, CA (pancreas), Diabetes Type II, Hypothyroidism, Hypertension	Compazine, Risperidone
20	F	81	14.5	N.A.	COPD, Pneumonia, Osteoporosis, Tuberculosis	*40*	M	55	12.6	N.A.	Schizophrenia, Paranoid Schizophrenia, Infection Bacterial, Psychotic disorder, Hallucination	Fluphenazine, Chlorpromazine

Abbreviations: ID: Identifier (progressive) number for each recruited subject; PMI: post-mortem interval; M: male; F: female; CA: carcinoma; COPD: chronic obstructive pulmonary disease; CVA: cerebrovascular accident; N.A.: Not Available.

Table S2. Comparisons of mRNA (expressed as relative quantification) and protein levels (expressed as % of control) between patients with schizophrenia and control subjects in the *post-mortem* dorsolateral prefrontal cortex and hippocampus, and between Alzheimer's or Parkinson's disease patients and control subjects in the *post-mortem* superior frontal gyrus. Adjusted p-values correspond to the raw p-values corrected for multiple comparisons using the Bonferroni-Dunn method; *p< 0.05.

		DLPFC	HIP	SFG	
		CTRL vs SCZ	CTRL vs SCZ	CTRL vs PD	CTRL vs AD
	mRNA/protein	p-value adjusted	p-value adjusted	p-value adjusted	p-value adjusted
mRNA	*CAP2*	0,073	0,034*	0,013*	0,122
	DLG1	0,041*	0,574	1,000	1,000
	ADAM10	0,397	0,752	1,000	0,462
Protein	CAP2	1,000	1,000	1,000	1,000
	SAP97	0,109	0,670	1,000	1,000
	ADAM10	0,964	1,000	1,000	0,968

Abbreviations: DLPFC: Dorsolateral prefrontal cortex; HIP: Hippocampus; SFG: Superior frontal gyrus; CTRL: Control subjects; SCZ: patients with schizophrenia; PD: Parkinson's disease patients; AD: Alzheimer's disease patients.
